# Modeling and analysis of the dynamic behavior of the XlnR regulon in Aspergillus niger

**DOI:** 10.1186/1752-0509-5-S1-S14

**Published:** 2011-06-20

**Authors:** Jimmy Omony, Leo H de Graaff, Gerrit van Straten, Anton J B van Boxtel

**Affiliations:** 1 Systems and Control group, Wageningen University, Wageningen, P.O. Box 17, 6700 AA, The Netherlands; 2 Laboratory of Systems and Synthetic Biology, Wageningen University, Wageningen, Dreijenplein 10, 6703 HB, The Netherlands

## Abstract

**Background:**

In this paper the dynamics of the transcription-translation system for XlnR regulon in *Aspergillus niger* is modeled. The model is based on Hill regulation functions and uses ordinary differential equations. The network response to a trigger of D-xylose is considered and stability analysis is performed. The activating, repressive feedback, and the combined effect of the two feedbacks on the network behavior are analyzed.

**Results:**

Simulation and systems analysis showed significant influence of activating and repressing feedback on metabolite expression profiles. The dynamics of the D-xylose input function has an important effect on the profiles of the individual metabolite concentrations. Variation of the time delay in the feedback loop has no significant effect on the pattern of the response. The stability and existence of oscillatory behavior depends on which proteins are involved in the feedback loop.

**Conclusions:**

The dynamics in the regulation properties of the network are dictated mainly by the transcription and translation degradation rate parameters, and by the D-xylose consumption profile. This holds true with and without feedback in the network. Feedback was found to significantly influence the expression dynamics of genes and proteins. Feedback increases the metabolite abundance, changes the steady state values, alters the time trajectories and affects the response oscillatory behavior and stability conditions. The modeling approach provides insight into network behavioral dynamics particularly for small-sized networks. The analysis of the network dynamics has provided useful information for experimental design for future *in vitro* experimental work.

## Background

The filamentous fungus *Aspergillus niger* is an important organism in the production of enzymes and precursors for the food and chemical industries. Industrial citric acid production by *A. niger* represents one of the most efficient, highest yield bio-processes in use by industry. The xylanolytic activator gene *xlnR* is a main controlling gene in the XlnR regulon of *A. niger*.

The XlnR regulon is activated by D-xylose in the culturing media [1]. The current description of this system is based on static interpretation of the system. Experiments [2] showed, however, that the expression of genes in the XlnR regulon is dynamic. Therefore, to advance the application of *A. niger* by better understanding of the XlnR regulon, the dynamics of the regulon needs to be quantified. For this purpose time course experiments are scheduled. However, planning of the experiments is improved by quantifying the behavior by a simulation and analysis study prior to the experiments.

For the XlnR regulon, literature information on the network structure was used as a basis for the simulations. To our knowledge, currently very little has been done on modeling the dynamics of the XlnR regulon and also on time course profiling of the genes that constitute the XlnR regulon in *A. niger*. The challenge with genetic network modeling lies with determining a specific equation formalism to represent the network structure. One of the suggested strategies of modeling using differential equations is to fix the form of the equation [3,4]. Prior knowledge on the network structure is essential to develop a quantitative model [5]. The descriptive information on the XlnR regulon [1] enables us to hypothesize models for the interaction between the different network components.

Generally, in the study of biological networks, positive feedback (PFB), negative feedback (NFB) [6-9], feedforward loops and time delay [10] have been shown to be influential. NFB loops cause oscillatory behavior if the signal propagation around the feedback loop is low and PFBs can lead to a bi-stability behavior [11]. Overall, feedback plays an important part in biological networks by allowing the cell to adjust to the repertoire of functional proteins to current needs. Other examples of biological systems in which the effect of feedback and time delay were extensively studied can be found in the developmental regulator *Hes1*, which inhibits its own transcription [12,13] or in the NFB loops in the p53 response [14,15]. Bliss *et al*. [16] investigated conditions on parameters that ensure stability of the unique steady states in an operon using differential equations modeling. These authors then chose parameters that allowed the model to describe the tryptophan operon of *E. coli*. Their investigations focus on network stability in steady state. They showed that with parameters corresponding to a mutant with reduced repression, stability conditions were violated leading to oscillations. The analysis of the condition on parameters that ensure steady state stability also lead to insight into direct repression of a gene by its own product [16].

In computational systems biology, numerous studies have been done on genetic network reconstruction using time course data but little attention has been given to understanding the network dynamics. It is crucial to understand or at worst have a fuzzy idea of a biological network dynamics if one is to gain deeper insight into the biological network dynamics and functionality.

This paper concerns the analysis of the network dynamic behavior, the effect of feedback loops and the conditions under which oscillatory responses in metabolite expression may be exhibited are investigated. Modeling of the XlnR regulon is explored by using nonlinear differential equations and Hill functions for the transcription and linear reaction kinetics for the translation process. To ensure that detailed aspects of the transcription-translation model formalism are captured, some assumptions are incorporated in the modeling. *In silico* perturbation experiments were performed by triggering the genetic network at steady state. The advantages of using ordinary differential equations (ODEs) are vast since they are capable of modeling degradation effects and causal effects in a network [17].

Applications of dynamical systems in modeling transcription regulatory networks can be found in [18-21]. Many of these studies used the continuous-time domain to model gene expression as biochemical processes using in ODEs. The modeling and analysis aims to identify which factors determine the dynamics to aid and guide future time course experimental studies. In our work, we highlight the need to understand the dynamics of biological networks with the hypothesis that modeling and experimentation should go hand in hand.

## Methods

### Regulation mechanism for the XlnR regulon

In the model organism *Aspergillus niger*, transcription of genes encoding xylanolytic and cellulolytic enzymes take place [1]. Activation enables the degradation of the cellulose and hemicellulose from plant cell walls. XlnR is a zinc binuclear cluster protein consisting of about 875 amino acids. It is suspected that XlnR binds as a monomer [1]. The *xlnR* gene is induced in the presence of D-xylose in the culturing media and repressed in the presence of the carbon catabolic repressor, CreA [22].

**Figure 1:**
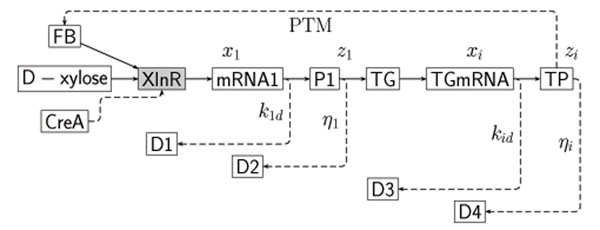
**The XlnR regulon scheme** The XlnR regulon induced by D-xylose in the presence or absence of CreA. The representations P1 and TP are the proteins from the *xlnR* gene and target genes, respectively. The terms mRNA1 and TGmRNA represent the transcription products from the *xlnR* gene and target genes, respectively. FB represents the feedback protein in which post-translational modifications might take place.

Gene regulation can take place at different stages of the central dogma of molecular biology (DNA→ RNA → Protein). These stages include among others transcription, translation and post-translational modifications (PTMs) of the associated protein. In Figure 1 a scheme of the activities in the XlnR regulon is given. The *xlnR* gene is induced by D-xylose. At induction the *xlnR* gene produces messenger RNA (mRNA) which is translated in proteins. These proteins then activate the target genes (TG). For the XlnR regulon, the number of target genes are estimated to be in the order of 20 to 40. In Figure 1 all target genes are represented by TG. After transcription and translation of the target genes, the target proteins (TP) are obtained. Protein from PTMs can be involved in the regulation of the *xlnR* gene trough a feedback loop. At each step in transcription and translation mRNA and proteins can be degraded and/or used for other processes (D1–D4).

### Transcription model

Commonly, hyperbolic functions and the sigmoid class of functions are used to represent the kinetics of gene regulation [23]. Such functions mimic the nonlinearity in gene regulation, by assuming that a critical amount of protein has to be accumulated before a gene can be considered regulated or repressed. The most common form of function used for modeling gene transcription is the Hill function [24,25].

Let the vector **z** = [*z*_1_,…,*z_n_*]*^Τ^* represent the concentrations of the translated proteins corresponding to the genes 1,…,*n*; where *n* is the number of genes involved. Throughout this work, the notation, e.g. *z_i_* is used to represent the time dependent variable *z_i_*(*t*) (where *t* ∈ [0, ∞)). Then the activating and repressing functions are given by(1)

where *ψ*^–^(*z_i_*, *θ_i_*) = 1 – *ψ*^+^(*z_i_*, *θ_i_*), *θ_i_* is the gene specific half-saturation parameter and the positive number *h* represents the Hill coefficient. The regulation mechanism for each target gene *i* is captured by the function Ψ(*z_i_*, *θ_i_*) in (1).

According to Hasper *et al*. [26] there is evidence that although most zinc binuclear cluster proteins bind as a dimer, it seems that XlnR binds as a monomer - therefore, a Hill coefficient with *h* = 1 is used. Given the availability of structural prior knowledge and that the master regulator activates the target genes, the nonlinear system that models the transcription process is given by(2)

where *k_i_*_1_ = 1/*θ_i_* for *h* = 1, *x_i_* - mRNA concentration from gene *i*, *ρ_i_* is the basal (or leaky) transcription rate for gene *i* and is associated with very low levels of mRNA, *k_i_*_1_ - effective affinity constant for gene 1 activating gene *i* (*i* = 2,…,*n*), *k_is_* - maximum synthesis parameter for gene *i*, *k_id_* - first order degradation rate (or consumption rate) for gene *i*, **x**_0_ - vector of initial mRNA concentration, *z_i_* - concentration of translated protein from gene *i*, **b** = [*b*_1_,…,*b_n_*]*^Τ^* is the input matrix and **u** = [*u*_1_,…,*u_n_*]*^Τ^* is the input vector (gene triggering compounds). The model formulation with no feedback later on aids the assessment of the marginal contribution of a feedback loop in the network dynamics.

### Translation model

A system of linear differential equations to model the protein abundance (translation process) is then considered. The linear model representations (3) are used to capture the dynamics of the translation process with both the production and degradation terms being linear.(3)

where *r_i_* - specific translation rate for gene *i*, *η_i_* - degradation rate for protein *i*. The *z_i_*’s represent the target proteins in the scheme in Figure 1. At steady state the response rate and degradation rate balance, i.e. *ẋ*_1_ ≈ *ẋ*_2_ ≈ … ≈ *ẋ_n_* ≈ 0. By setting the transcription rates *ẋ*_i_ = 0 for all *i* in (2), it follows that(4)

The steady state values in (4) are based on the assumption that, for a small time window the change in concentration of the input stimulus and metabolite concentrations remain nearly unchanged. The model specifications for the transcription and translation process describe the rates of change of concentration of the genes and proteins. Overall, the system of 2*n* coupled differential equations in (2) and (3) describe the network dynamics.

### System stability

The interesting case to analyze is the systems behavior in the absence of the inhibitor, CreA. Let us denote the equilibrium concentrations of mRNA and protein quantities by the vectors  and  respectively. Using (3) the steady states lead to the relationships  for all *i*. The stability of each steady state (from (2) and (3)) can be analyzed using Hopf Bifurcation, an analytic approach that has been widely used in investigating stability conditions in gene expression networks [27-30].

Let *F* : ℝ^2*n*^ → ℝ^2*n*^ be a set of smooth functions (with *F* = (*F*_1_,…,*F*_2*n*_)) that capture the XlnR regulon system dynamics. In this case we have *F*_1_ = *ẋ*_1_,…,*F_n_* = *ẋ_n_*, and *F*_*n*+1_ = *ż*_1_,…,*F*_2*n*_ = *ż_n_* in (2) and (3) respectively. By definition, the Jacobian matrix is given by(5)

This Jacobian matrix is used to assess the regulon stability and to identify which parameters dictate the transcript abundance. First, consider a case of three genes and three proteins, *n* = 3. The Jacobian is given by(6)

where **x** = [*x*_1_, *x*_2_, *x*_3_]*^Τ^* and **z** = [*z*_1_, *z*_2_, *z*_3_]*^Τ^* . The corresponding steady states of the vectors  and  can be computed, accordingly. Using expressions (2) and (3) in (5) we obtain(7)

where(8)

for *i* = 2, 3. A similar generalized expression for  can be obtained given a regulon with a known number of transcripts *n*. The characteristic polynomial obtained from (7) is given by(9)

In the case of this regulon, the derived characteristic polynomial turns out to be the same as the determinant, i.e. . Using the expression (9), conditions that ensure global stability can be established on the parameters.

The formulations of the Jacobian matrix and the eigenvalue spectra can be extended to an *n*-dimensional network system. The generalization for the eigenvalue spectra using a similar analysis as above leads to the expression(10)

for *n* ∈ ℤ^+^, a positive integer. In the network without feedback, it turns out that the trace of the Jacobian matrix is equal to the sum of all the eigenvalues (i.e.  holds true). The above analysis shows that for the open loop system there is no possibility for oscillatory behavior to occur, and that the time constant only depends on the degradation coefficients.

According to Aro *et al*. [31], van Peij *et al*. [1] and Hasper *et al*. [32], the *A. niger* genes *eglA*, *eglB*, *eglC*, *cbhA*, *cbhB*, *xlnB*, *xlnC* and *xlnD* contain binding sequences (GGCTAAA) to the XlnR protein as well as binding sequences to CreA, a repressor protein acting in the presence of monomeric sugars (i.e., glucose) as an auto-regulating mechanism. This property ensures that most target genes have similar expression dynamics in time.

### Feedback in the network

Numerous transcription systems are known to include genes that regulate their own expression values [33]. In our analysis, to model the effect of feedback we hypothesize that the TPs and PTMs in the feedback loop in the scheme in Figure 1 only act on the *xlnR* gene. Therefore, only the equation that captures the dynamics for the first gene (*x*_1_) has to be modified accordingly. The adapted equation is given by(11)

where(12)

is the repressor Hill function and *C_A_* - quantitative activity state for CreA, *k_A_* - inverse of the Hill constant of CreA. The term *τ* > 0 represents a time delay in the feedback loop. The sets **S**_1_ = {*j* | *j* = 1,…,*m*} and **S**_2_ = {*l* | *l* = *m* + 1,…,*n* – 1} where **S**_1_ ⋃ **S**_2_ = {1,…,*n* – 1} i.e. collection of all the target proteins in the regulon. All the supposed repressing and activating proteins are lumped in the sets **S**_1_ and **S**_2_, respectively. The effect of the D-xylose and the feedback loop is modeled as additive. Equation (11) also specifies the build up of proteins and repression or activation of the *xlnR* gene through the feedback loop. Through the sequence of PTMs the protein availability in the feedback loop is delayed. All the other components representative of the target genes in the network models (2) and (3) remain unchanged.

#### xlnR gene promoter activity under feedback

Let us define the promoter activities by the expressions (13) and (14). The promoter activity corresponding to the case when an activating protein is involved in the feedback loop is represented by the term Γ_A_ and that for the case of a repressing feedback effect denoted by Γ_R_.(13)(14)

The extracts from the denominator functions are given by (15) and (16), respectively.(15)(16)

These terms are used in the calculations for the activating and repressing promoter activity for the XlnR regulon. For the sake of illustrations, two target genes were considered (i.e. values of *j* = 1 and *l* = 2) in the simulation with one as an activator and the other as a repressor (the values *k_RL_* = *k_AL_* = 1 were used). We consider the sets **S**_1_ and **S**_2_ of unit elements which index the proteins that are responsible for regulating the *xlnR* gene through a series of mechanisms in the PTM channel.

#### Existence of oscillatory behavior

The eigenvalue spectra from the derived Jacobian matrix can be used for this analysis. The presence of at least a pair of eigenvalues with complex parts implies the existence of oscillatory behavior. The PTMs in the feedback loop may produce oscillatory behavior depending on the individual attributes of the target genes and the consequent proteins in the feedback loop.

We observed that in the absence of a feedback loop, the system dynamics is dictated by the degradation parameters. Active degradation of proteins or mRNA is a major part of many metabolic and stress response systems [11]. This may not necessarily hold true for the system with a feedback loop because of the structural change in the Jacobian matrix. The metabolites involved in this oscillatory dynamics are presumably determined by the individual biochemical and mechanistic attributes of the individual molecules. Therefore, no single hard rule for classifying which metabolites are responsible for the overall oscillatory behavior of the expression profiles of the genes and proteins can be stipulated. This might however be possible for some specific network pathways for which extensive information is available. Consider the Jacobian matrix corresponding to three genes and three proteins (17). We now analyze the effect of having a protein in the feedback loop. These proteins are considered to regulate the *xlnR* gene and their possible positions in the Jacobian matrix are indicated by ”×” as shown in (17).(17)

Using the adapted model (11), the computed entry in the (1, *n* + *i*)-th cell (*i* = *j*, *l* for all values of *j* and *l*) of the Jacobian matrix (5) is given by (18) and/or (19) depending on which proteins in the feedback loop are involved in the regulation of the *xlnR* gene. By taking partial derivatives of the function *F*_1_ with respect to the variable of interest within each of the sets **S**_1_ and **S**_2_, we then have the more compact expression(18)

This term corresponds to the repressing proteins, and the terms given by(19)

for the activating proteins. The parameters *k_AL_* and *k_RL_* represent the lumped affinity constants for the activating and repressing proteins, respectively. Suppose that the XlnR protein (*i* = 1) is the only metabolite responsible for auto-regulation in the feedback loop. By representing the corresponding entry at the (1, 4)-th position in the matrix (5) by a nonzero parameter *ω*_1_ ∈ ℝ\ {0}, a parameter that intrinsically represents the auto-regulation effect of the *xlnR* gene. The computed eigenvalue spectrum from (7) using the expression  is given by(20)

where(21)(22)

are the conjugate roots. The eigenvalues *λ*_5_(·) and *λ*_6_(·) may take on values from the real space, ℝ or the complex space, ℂ. From (21) and (22) we observe that oscillation can only be obtained if the condition *ω*_1_ < –(*η*_1_ – *k*_1*d*_)^2^/4*r*_1_ for *r*_1_ > 0 is satisfied. This condition on *ω*_1_ signifies a contribution from a NFB loop in the XlnR regulon network. Notice that the expression (*η*_1_ – *k*_1*d*_)^2^ > 0 for all values of *η*_1_ and *k*_1*d*_. This finding adds to consolidate the findings by Tiana *et al*. [15] from a theoretical analysis of three eukaryotic genetic regulatory network in which they attributed the existence of oscillation to a common design of a NFB with underlying time delay. Considering the expressions for the eigenvalues in (21) and (22), we observe that for a PFB effect of the XlnR protein there is no possibility for oscillatory behavior. This result does not necessarily hold true for the proteins in the feedback loop corresponding to the cells at positions (1, *n* + *i*) for *i* = 2,…,*n*.

The presence or absence of oscillatory behavior is insufficient for drawing conclusions about stability in system responses. Stability, using (21) and (22) exists if the conditions Re(*λ*_5_(·)) < 0 and Re(*λ*_6_(·)) < 0 are simultaneously fulfilled. Hence, the inequality(23)

Solving the inequality (23) for *ω*_1_ leads to the condition *ω*_1_ <*η*_1_*k*_1*d*_/*r*_1_. A similar analysis for the existence of oscillatory behavior and stability dynamics can be done for the other proteins in the feedback loop, for example at position (1, 5) and (1, 6) or combinations in the matrix (17). However, although such analysis is conceptually simple, the analytic expressions are very complex to work with. Information about the stability and oscillatory behavior is obtained by numerical solutions. An example is considered to investigate the time evolution of gene activity and protein abundance in the XlnR regulon.

#### Bifurcation analysis

Bifurcation analysis relates to stability on the system parameters. Stability properties for the system without feedback are given by (10), where it was shown that the roots of the characteristic polynomial correspond to the degradation rate constants for the mRNA expression and protein abundance. As these constants are positive, the system is always stable. In the case that one of them equals to zero, then the system is critically stable.

For the network with feedback loop, consider the conjugate roots in (21) and (22) denoted by *λ_i_*(·) for *i* = 5, 6. In the event that the conditions |Re(*λ_i_*(·))| = 0 and | Im(*λ_i_*(·))| ≠ 0 are simultaneously fulfilled - then there exists a Hopf bifurcation for the corresponding genes and proteins. Such a bifurcation occurs when the root of the positive discriminant function in (23) equates to the sum of the degradation parameters for the *xlnR* gene and XlnR protein, i.e.(24)

or after working out becomes *ω*_1_ = *η*_1_*k*_1*d*_/*r*_1_. This example illustrates the case of a feedback in the cell at position(1, 4) of the matrix in (17). The analysis for the other entries of *ω* results in highly complex expressions, therefore a numerical analysis is preferred.

## Results

### System specification

The analysis is illustrated by an example case. Consider a regulon network of three genes given a perturbation of D-xylose. The pulse perturbation takes place at time *t* = 0. During fermentation, the D-xylose is consumed and the D-xylose concentration follows the expression *u*(*t*) = *u*(0)(1/(*β* + *e^Kt^*)), where *u*(*t*) ≡ [D-xylose] and *β* > 0, with *K* = 0.3 and *u*(0) = 50 mM as the initial D-xylose concentration. The parameters used for the simulation are: *b*_1_ = 1, *ρ*_1_ = 2*e* – 3, *ρ*_2_ = 2.5*e* – 3, *ρ*_3_ = 1*e* – 3, *k*_1*d*_ = 0.5, *k*_2*d*_ = 0.4, *k*_3*d*_ = 0.3, *k*_2*s*_ = 5, *k*_3*s*_ = 6, *k*_21_ = 0.1, *k*_31_ = 0.1, *r*_1_ = *r*_2_ = *r*_3_ = 0.5, *η*_1_ = 1, *η*_2_ = 1 and *η*_3_ = 1.

### Stability and response analysis - without feedback

The expression for the characteristic polynomial, ***P***(·) in (10) is independent of the translation rate parameters *r_i_*, the gene synthesis coefficient *k_is_* and the terms in the expression (8). From (10) it can be observed that without feedback, the system is globally stable (i.e. the conditions  and  are satisfied for all  and  ). The system stability behavior is dictated by how fast the translation and transcription rates are (i.e. magnitudes of *k_id_* and *η_i_*).

**Figure 2:**
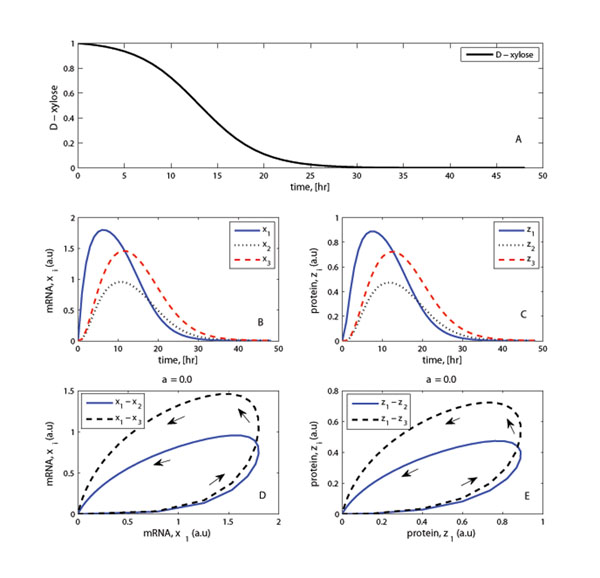
**D-xylose consumption, gene expression, protein abundance and phase plots: without feedback** (A): The simulated trajectory for D-xylose consumption. (B): Gene expressions profiles. (C): Proteins abundance plots. (D): Phase plot for gene expression showing variation of mRNA concentrations of the *xlnR* gene and the other target genes, *x_i_*. (E): Corresponding protein abundance phase plot.

In Figure 2 both the gene expressions in plot (B) and protein abundance plot (C) show similar behavioral dynamics. Moreover, with the chosen input pattern of D-xylose the target genes show phase plots similar in patterns but with variations that are dictated by individual gene or protein kinetic parameters. A relaxation time of *τ*_*R*1_ = 1/*k*_1*d*_ ≈ 2 hours is noticed for the master regulator and for the target genes, *τ*_*R*1_ <*τ*_*R*2_, *τ*_*R*3_. The relaxation time is an approximation for the time required for the system to relax into steady state. This represents the time it takes a system to react to a persistent external input (D-xylose).

### Feedback in the network

Since the presence of CreA is a strong repressor that inhibits the *xlnR* gene activity by blocking the promoter binding site, we chose to model this influence by considering a switch-like function with *H* ∈ {0, 1}. Here the assignment of *H* = 0 and *H* = 1 means CreA is present and absent respectively. In the absence of CreA the protein products from the target genes are involved in regulating the activity of the master regulator. These protein products may either inhibit or activate the *xlnR* gene.

**Figure 3:**
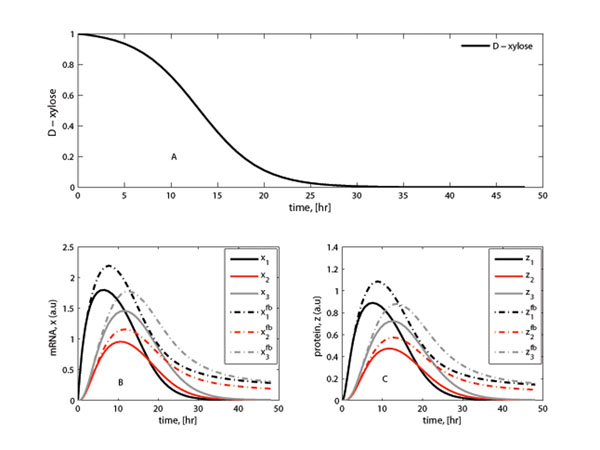
**D-xylose consumption, gene expression, protein abundance and phase plots: with feedback** (A): The simulated trajectory for D-xylose. (B): Gene expression profiles with solid lines (–) showing the expression profiles for the genes in the absence of CreA. The corresponding dotted lines (⋯) show the simulated effect of competitive feedback (with *τ* = 1). (C): Protein abundance profiles (solid lines).

A comparison of the metabolite expression dynamics for the network with and without feedback loops in the absence of CreA is shown in Figure 3. The same parameter values in the section **System specification** were used for the simulation with the extra parameters from (11) being *k_RL_* = 1 and *k_AL_* = 1 and the lumped synthesis parameter from (11) chosen as *k_ls_* = 1. Figure 3 indicates the enhanced metabolite expression as a result of incorporating a feedback loop with delay (with *τ* = 1) in the model - a result that is similar to what was observed by Maithreye *et al*. [*34*] during a theoretical kinetics analysis of the concentration of green florescent protein (GFP) in time.

#### Activating and repressing feedback

The expressions (18) and (19) have the potential to yield oscillatory behavior in the metabolite response profiles. The oscillatory behavior (if and when it exists) is purely governed by the values of the system mechanistic parameters. Such oscillatory behavioral patterns of gene expression may vary from organism to organism, and can be detected from time series data if enough samples are taken.

**Figure 4:**
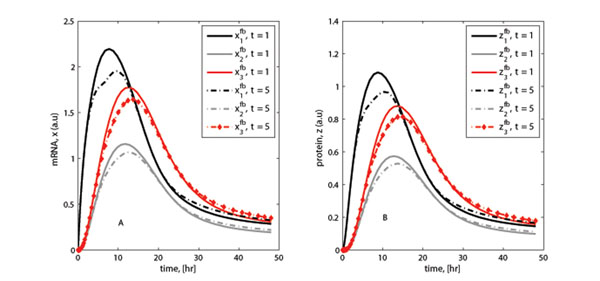
**Effect of time delay on expression** (A)-(B): Plots showing the effect of variation in time delay in the feedback loops corresponding to the transcription and translation processes, respectively. The observed effect on the responses is small except for the slight deviation at the peak of the expression profiles.

To assess the effect of time delays in the transcription and translation processes, some cases were simulated. The results of the expression time-dynamics for both the genes and proteins are shown in Figure 4. The simulations were performed for specific cases of *τ* = 1 hour and *τ* = 5 hours and the subsequent outputs compared. The metabolite expression patterns from the two cases are nearly similar with the main difference occurring at the maximum level. Overall, longer time delays lead to a small and non significant reduction in expression values.

#### xlnR gene promoter site activity

**Figure 5:**
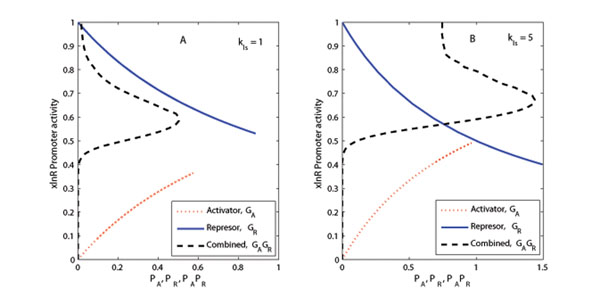
**xlnR promoter region activity** Plot of the *xlnR* promoter region activities defined by Γ_A_, Γ_R_ and Γ_A_Γ_R_ depending on the regulator. The term Γ_A_Γ_R_ - is the combined affect of competitive binding to promoter region by activators and repressors. Plots (A) and (B) show the influence of weak (*k_ls_* = 1) and strong (*k_ls_* = 5) synthesis parameters respectively.

The competitive effect of the activators and repressors for the promoter binding sites was also simulated. The effect of which transcription factor (TF) (either an activator or a repressor) wins occupancy of a promoter binding site depends partly on the strength of the synthesis parameter *k_ls_* (Figure 5).

The promoter is most active (activity around 50 – 80%) when the regulon is fully active. This corresponds with the time window at which the network is fully responsive to the external perturbation. We observe that the *xlnR* gene activator has a tendency of occupying most of the promoter sites at any given time (Figure 5).

#### Bifurcation and oscillatory behavior analysis

**Figure 6:**
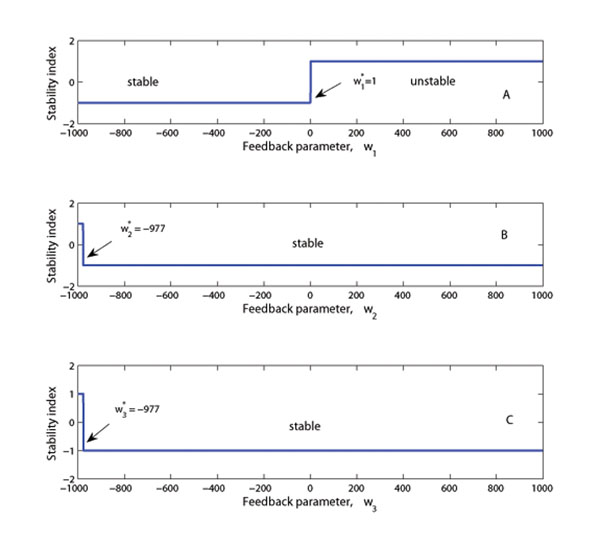
**Stability index curves** (A)-(C): Plots of the stability indices for various values of *ω_j_* ∈ (–1000,1000) for *j* = 1, 2, 3. A stability index value of –1 and +1 indicates stable and unstable responses of gene and protein expression in time, respectively. The value *ω_j_* < 0 represents repressing feedback and *ω_j_* > 0 is the activating feedback effect. Simulations performed using parameters from **System specification** and the pseudo steady state expression values at which the Jacobian is estimated.

A range of values of activating and repressing parameters *ω*_1_, *ω*_2_, *ω*_3_, respectively on entries (1, 4), (1, 5) and (1, 6) in expression (17) was considered for analyzing the stability behavior of the network. It was observed that NFB on *ω*_1_ gives a stable system and values of *ω*_2_ and *ω*_3_ below –977 results in an unstable systems, Figures 6 (A)-(C). The PFB effect of the XlnR protein on the *xlnR* gene leads to unstable system dynamics for *ω*_1_ > 1. This can be seen from Figure 6 (A). This result also leads to the conclusion that the XlnR regulon is unstable if the *xlnR* gene has a PFB from its own protein.

**Figure 7:**
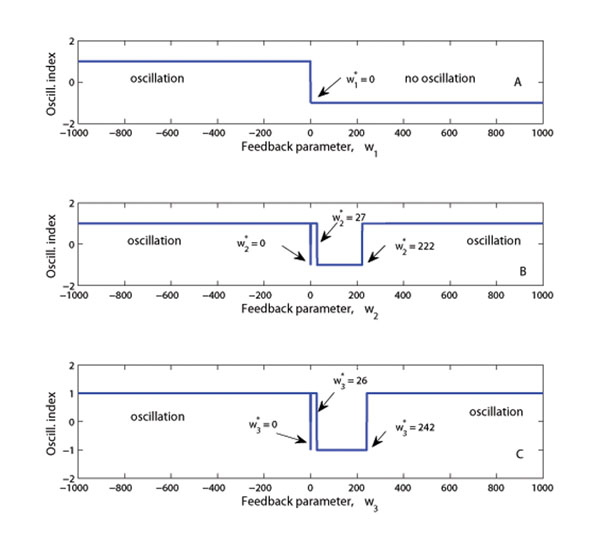
**Oscillation index curves** (A)-(C): The plots indicate possible oscillatory behavior for various values of *ω_j_* ∈ (–1000,1000) for *j* = 1, 2, 3. Each *ω_j_* is considered independently while the others are set to zero. The oscillation indices –1 and +1 represent non-existence and existence of oscillatory behavior in the response behavior of the metabolites, respectively.

An analysis of how the various feedback parameters affect the oscillatory behavior of the gene and protein expression was also considered. The results show that there exist threshold values (or a range of parameter values) for the feedback parameters *ω_j_*’s for which their oscillatory behavior may or may not occur (see Figure 7 (A)-(C)). The value *ω_j_* = 0 corresponds to no feedback in the system and according to the previous analysis (under the subsection: stability and response analysis - without feedback), no oscillation occurs in this case. A transient oscillatory behavior is observed for values of the parameter *ω_j_* ≈ 0 for all *j*, Figure 7 (B)-(C). The observed stability curve corresponding to the XlnR protein in the feedback loop (*ω*_1_) is a near reflection of the corresponding resultant oscillation curve (Figure 6 (A) and Figure 7 (A)).

## Discussion

The model gives a better understanding of the rate limiting steps in the process of activating the XlnR regulon and therefore, helps to define the biological control points. Similarly this knowledge can be used to obtain strains that have enhanced xylanolytic enzyme production. These enzymes are industrially of importance as food and feed additives, but are also part of a system that is used to bleach paper pulp. Given that the transcription rate and degradation rates have been shown to be the key parameters that dictate the systems dynamics for the XlnR regulon; this information is important for designing and sampling of time course experiments. Once the transcripts are unstable, the proteins get quickly degraded; otherwise they remain stable. This observation is linked to the D-xylose uptake in fermentation experiments. The consumption of D-xylose also indirectly controls the regulation of the target genes and therewith the breakdown of sugars.

Simulations showed that the dynamics of the D-xylose input function considered in the examples has an important effect on the profiles of the individual metabolite concentrations. This is particularly dictated by the value of the parameters in the external input function *u*(*t*). The larger the value of the *K*, the faster the consumption of D-xylose. This depends on the chemical reactions taking place in any given cell, or the saturation levels of the individual compounds in a cell.

Feedback significantly affects the response of the output profiles for the metabolites and changes the final steady state values (Figure 3). Further simulations showed that variations of the time delay in the feedback loop (*τ* = 1, 2,…, 5 hours) have a small effect on the pattern of the response (Figure 4). The stability analysis subsection shows that the metabolite response dynamics exhibits no oscillatory behavior for a network without feedback loop. For a network with feedback loops, the results from numerical analysis showed that feedback conditions for which the system is stable or for which the system exhibits oscillatory behavior can be obtained (Figure 7 (A)-(C)). In modeling the feedback loop, time delay was accounted for and included in the model.

According to Bliss *et al*. [16] and Thomas and d’Ari. [35], including time delay in modeling biological networks is considered important because many biological systems exhibit some delays in their feedback loops. However, according to our finding (Figure 4), incorporating the time delay had no strong effect on the overall dynamics of the metabolite expression profiles.

The analysis shows that the existence or absence of oscillatory behavior is dictated by the numerical values of the individual mechanistic parameters. The conditions for oscillatory behavior follow from the eigenvalue spectra. The eigenvalue spectra analysis like that in (20) and the corresponding conditions for which all the eigenvalues are less than zero, gives also indication for the stability properties for the XlnR network with feedback loop. If all the eigenvalues satisfy the condition  for all entries, then the system is stable, otherwise it is unstable.

Two scenarios can be considered: one in which the proteins involved in the feedback loop are activating and the other in which the proteins are repressing. The details of the expected behavioral dynamics from such a system requires a case by case analysis (like in Figure 6 (A)-(C)) of the effects of the proteins in the feedback loop. A similar analysis can be extended to study the stability in case of a combined effect of any two or more proteins of interest. When the number of network components become large, obtaining explicit analytic solutions and expressions for the eigenvalues and other quantities of interest increasingly become complex - in which case the alternative of numerical methods can be used (see Figure 6 (A)-(C) ). Thomas and d’Adri [36], and Thomas *et al*. [35] investigated the properties of mathematical Boolean net Modeling Genetic Networks works - investigations that provided significant insight into genetic network dynamics. In their work they showed the importance of NFB loops for maintaining homeostasis in levels of gene products. Our analysis leads to the observation that having a NFB loop stabilizes the response of the metabolite expressions (Figure 6 (A)-(C)). However, there exists certain ranges of values of the strength of feedback effects that make the system unstable. This sets constraints to the feasible parameter for the system if instability is not observed. In some cases having a PFB loop yields a stable network response, Figures 6 (B) and (C). This result is in agreement with that found in a study by Maithreye *et al*. [34]. In their investigations they found that NFB loops provide stability and withstand considerable variations and random perturbations of biochemical parameters.

The effect of time delay on stability can be analyzed from a transfer function of the model in the ”*s*” domain, or by a transformation to the ”*z*” domain. In these cases the delay time is considered as a finite dimensional system. Stability analysis can be done by searching for stability properties in the ”*s*” domain or ”*z*” domain. Examples of other methods that deal with the delay times are given for state estimation in the work by Liu *et al*. [37] and Yu *et al*. [38].

The adaptive filtering approach developed in [38] is based on the adaptive synchronization setting, for estimating unknown delayed genetic regulatory networks with noise disturbance. Using this approach, no exclusive knowledge of system parameters is required, e.g. those lacking in the XlnR regulon and many other biological networks. Liu *et al*. [37] proposed an adaptive feedback control approach for simultaneously identifying unknown (or uncertain) network topological structure, unknown parameters of uncertain general complex networks with time delay from available mRNA data and estimation of protein concentration. The effectiveness and applicability of their approach was shown using *in silico* numerical simulations. In contrast to [37] and [38], we study the XlnR regulon dynamics and do not focus on the system structure identification and parameter estimation.

According to Balsa-Canto *et al*. [39], powerful mathematical analytic tools highlight the value for successful study of many biological systems. However, such success can mainly be attributed to the unrelenting endeavors for an in-depth understanding of both computational methods and the biological problems of interest. For the case of the XlnR regulon, our analysis provides a basis for understanding the behavioral dynamics of genes and proteins after network perturbation. This will form a basis for future wet-lab experiments, particularly with the genes from the XlnR regulon. Given that the metabolite expression dynamics are known, this study provides a basis for strategic thinking in line with experimental design. The modeling approach used in this paper provides good information for understanding network behavioral dynamics particularly for small-sized networks. This is illustrated by the XlnR regulon in which even the simplest of structures can yield interestingly complex dynamics. Therefore, a reasons for having limited our focus to the regulon dynamics. Having detailed information regarding the basal parameters and the other mechanistic parameters might further improve the analysis and investigations into the network dynamics. Nevertheless, with informed parameter guesses, simulation studies provide good information into the systems behavior.

## Conclusions

The investigations in this paper considers the XlnR regulon as a dynamic system instead of a static system. Our study provides insight into the dynamic properties of the XlnR regulon. By studying this system, it has become more clear that the transcription and translation degradation rate parameters and the D-xylose consumption profile dictate most of the dynamics in the regulation properties of the network. The existence of oscillatory behavior depends on the conditions of the mechanistic parameters in the feedback loop - conditions that cannot always be generalized analytically and therefore, must be treated by numerical analysis. The role played by feedback in the network dynamics was found to be significant on the expression dynamics of genes and proteins. This means that the effect of the feedback should be considered in the model if there is sufficient supportive biological need or evidence from data. Just like for most biological systems, this is no doubt an important piece of information for the accurate modeling of biological network.

The analysis of the network dynamics has provided useful information for future *in vitro* experimental work. Particularly the potential for hypothesis testing basing on this work and the design of related perturbation experiments to generate time series data. Once there are available techniques for the network structural identification and parameter estimation for the XlnR regulon can be investigated.

## Authors’ contributions

LHdG provided the biological knowledge that was used for the model formulations. JO performed the modeling, data analysis and wrote the manuscript. GvS and AJBvB also contributed in calculations and critical review of the methods used in the analysis. All authors read and approved the final manuscript.

## Competing interests

The authors declare that they have no competing interests.
